# Temporal patterns of owner-pet relationship, stress, and loneliness during the COVID-19 pandemic, and the effect of pet ownership on mental health: A longitudinal survey

**DOI:** 10.1371/journal.pone.0284101

**Published:** 2023-04-26

**Authors:** Niwako Ogata, Hsin-Yi Weng, Locksley L. McV. Messam

**Affiliations:** 1 Department of Veterinary Clinical Sciences, College of Veterinary Medicine, Purdue University, West Lafayette, Indiana, United States of America; 2 Department of Comparative Pathobiology, College of Veterinary Medicine, Purdue University, West Lafayette, Indiana, United States of America; 3 Section: Herd Health and Animal Husbandry, School of Veterinary Medicine, University College Dublin, Dublin, Leinster, Ireland; Universidade do Porto Instituto de Biologia Molecular e Celular, PORTUGAL

## Abstract

The COVID-19 pandemic has affected us in numerous ways and may consequently impact our relationships with pet dogs and cats. We conducted a longitudinal survey to examine the temporal patterns of owner-pet relationship, stress, and loneliness during four phases of the pandemic: 1) pre-pandemic (February 2020), 2) lockdown (April to June 2020), 3) reopening (September to December 2020), and 4) recovery (January 2021 to December 2021). We also investigated the effect of pet ownership on stress and loneliness, by considering a set of *a priori* causal assumptions. In addition, we hypothesized that the differences in the levels of stress and loneliness between dog and cat ownerships were mediated by the owner-pet relationship. A total of 4,237 participants (657 non-pet owners, 1,761 dog owners, and 1,819 cat owners) completed between one and six surveys. Overall, the closeness in the relationship between owners and their pets increased with time during the study period. We also observed that dog owners consistently showed larger decreases in the levels of stress and loneliness than cat and non-pet owners. However, after adjusting for confounders, the findings did not support a mitigating effect of pet ownership. Pet ownership did not alleviate stress, social loneliness resulting from a lack of friendships or workplace relationships, or emotional loneliness due to deficiencies in family relationships. Pet owners, however, reported a lower degree of emotional loneliness caused by deficits in romantic relationships than non-pet owners. Our results also indicated that the differences in stress and loneliness levels between dog and cat ownerships were partially explained by the owner-pet relationship, and once this was accounted for, the differences between them reduced. In summary, this study highlights the dynamic effects of COVID-19 on owner-pet relationship and mental health. It also shows the complexity of the association between pet ownership and mental health, partially mediated by owner-pet relationships.

## Introduction

Since the World Health Organization declared COVID-19 a pandemic on March 11th, 2020, many countries, including the United States, have experienced several distinct stages from the initial lockdown to prolonged recoveries and re-emergences. These different phases of the pandemic, each represent unique challenges. This provides an opportunity to investigate its dynamic effects on owner-pet relationship and mental health. For example, lockdown policies instituted might lead to social isolation, whereas periods of recovery and re-emergence might act as stressors due to uncertainty. A number of theories exist surrounding pet ownership, owner-pet relationship, and their associations with mental health [1–6]. The scale and extent of the pandemic provide an opportunity to investigate their relevance in unusual circumstances. For example, are the biophilia and attachment theories of why humans are attracted to animals still relevant during a global health crisis? Studies have shown an increase in demand for non-human animals and green space during the lockdown phase of the pandemic, suggesting that the COVID-19 mitigation measures might have influenced biophilia [7, 8]. In addition, how is the social support theory of the effect of companion animals, such as dogs and cats, on mental health to be interpreted given lockdown policies that discouraged or prevented social contact? For instance, owners might have spent more time at home and interacting with their pets during the lockdown. These changes in owner-pet interactions might compensate for the reduction in social contact with friends or colleagues. Circumstances changed again, from the lockdown to the recovery phase, when owners returned to work. These changes could have impacts on the behaviors of pets, owner-pet relationships, and ultimately the mental health of pet owners.

Although several studies have investigated the association between pet ownership and mental health during the pandemic, most are cross-sectional, have only collected data at one time early in the pandemic, and none have assessed the changes in owner-pet relationships over time [9–15]. Furthermore, conflicting results on the association between pet ownership and mental health have been reported [16–20]. A systemic review by Kretzler et al. revealed that the most profound discrepancies were between qualitative studies and quantitative studies [19]. The findings of two mixed-methods studies also support the notion that beneficial effects of pets on mental health were observed in qualitative investigations more often than in quantitative ones [15, 16]. In addition, the differences in approaches to measure and quantify owner-pet relationships, mental health, and the association between them, might also explain contradictory findings among quantitative studies.

The relationship between humans and companion animals is complex and multi-dimensional. The characteristics of the owners, the pets, and their interactions together determine the quality of the owner-pet relationship [21–23]. Adding to the complexity, are the contextual effects of their shared environment, including major life events such as the COVID-19 pandemic [24, 25]. The assumptions about the underlying causal structure connecting these parameters are necessary for the proper evaluation of confounding when making inferences from observational studies, such as in this study. Thus, we constructed a directed acyclic graph (DAG) to organize and communicate our causal assumptions [26, 27]. A DAG is a type of diagram used to graphically depict assumed causal relationships between study variables [28]. In creating our DAG, we used assumptions based on the biophilia, attachment, and social support theories [5, 6], on knowledge of the owner-pet relationship and the effect of pet ownership on stress and loneliness derived from empirical studies as well as on our expertise [2, 4, 29–32].

The objectives of this longitudinal study were to characterize the temporal changes in the relationship between owners and their pet dogs and cats across different phases of the Covid-19 pandemic and to examine whether pet ownership mitigated adverse effects of the pandemic on mental health, including perceived stress and loneliness. We hypothesized that pet owners would report lower degrees of closeness to their pets during the initial lockdown stage due to the sudden disruption and changes in routines but progressively higher levels of closeness as they spent more time with their pets during the pandemic. We motivated this hypothesis by previous findings suggesting that both sudden changes in routines and disruption are known to be the risk factors for pet behavior problems and relinquishment [30, 33]. In addition, we examined the association between pet ownership and stress and loneliness with the hypothesis that pet owners would have lower stress and loneliness levels than non-pet owners and that the magnitude of the mitigation effect of pet ownership was stronger with dog than with cat ownership, as suggested by previous studies [16, 34, 35]. Finally, we investigated whether any differences in the levels of stress and loneliness between dog and cat ownerships were mediated by the owner-pet relationship.

## Materials and methods

### Recruitment and cohorts

This study was reviewed and approved by Purdue University Institutional Review Board (IRB-202-760). Study participants were recruited and followed via an online crowdsourcing platform CloudResearch^®^, (formerly TurkPrime; Prime Research Solutions LLC) [36]. CloudResearch^®^, primarily designed for behavioral research and longitudinal studies, allowed us to efficiently and safely recruit and survey eligible participants without requiring person-to-person contact. We were also able to invite those who completed the initial survey to participate in the follow-up surveys without collecting personally identifying information (e.g., name and other contact information). Studies have shown that data collected via a crowdsourcing platform were equivalent or superior in quality to data collected from various other sources, such as student subject pools and professional panel data [37, 38].

Eligible participants were U.S. residents, who were 18 years and older, and who either had one or more dogs and/or cats but no other types of pets (e.g., reptiles, fish, or other exotic pets) or did not have any household pet at the time of completing the initial survey (i.e., recruitment). For participants who had more than one dog and/or cat, we asked them to answer the pet-related questions for the pet that they felt most attached to. Participants each filled out one initial survey, and based on their reported ownership status and selection of the pet for answering the survey, were divided into three groups: no-pet, dog, and cat groups. Based on the *a priori* sample size calculation, we aimed to recruit 250 non-pet owners, 600 dog owners, and 600 cat owners. This sample size would allow us to detect a standardized effect size (i.e., difference in means divided by standard deviation) of 0.30 at a confidence level of 95% and power of 90%. However, the sample size did not account for adjustment of confounders.

The initial cohort was established in June 2020, and at the time of the survey, the data pertaining to the pre-pandemic (February 2020) period and part of the lockdown (April 2020) phase were also collected retrospectively. Follow-up surveys were conducted in September 2020, and January, April, August, and December 2021. We recruited additional cohorts subsequent to the January 2021 survey to make up for attrition from the earlier cohorts and maintain the initially planned sample size for each group. Study participants were followed until they either reported a change in their pet ownership status or were lost-to-follow-up. The study period was divided into four different phases of the COVID-19 pandemic: 1) pre-pandemic—February 2020, 2) lockdown—April to June 2020, 3) reopening—September through December 2020, and 4) recovery—January through December 2021. The divisions of the phases were based on the major milestones of the COVID-19 pandemic (https://www.cdc.gov/museum/timeline/covid19.html, Accessed on 1/15/2023), and each represented distinct challenges for pet owners. The pre-pandemic phase refers to the period before March 2020 when the WHO declared COVID-19 a pandemic. The lockdown phase refers to the period when most states of the United States implemented lockdowns, curfews, or stay-at-home orders. The reopening phase refers to the period when the majority of the U.S. states lifted the lockdown or stay-at-home orders. The recovery phase refers to the period of wide distribution of COVID-19 vaccines to the public in the United States and reemergence of SARS-CoV-2 variants. COVID-19 vaccines were first available to U.S. residents in December 2020.

### Study surveys

The study surveys included questions about the participants (e.g., demographics, highest level of education, and annual household income), pandemic-related concerns and practices (e.g., high-risk individuals, lockdown policy, social distancing, mask-wearing, vaccination, perceived risk of COVID-19 transmission between humans and pets, and COVID diagnosis), major life events (e.g., changes in employment and residence), and environment (e.g., housing and other household members). The personality of the participants was also assessed using the Five-Factor Model Rating Form, which consists of the five broad domains of neuroticism, extraversion, openness, agreeableness, and conscientiousness [39].

The surveys also included a series of questions about the characteristics of the pet and pet ownership, including the species, breed, sex, weight, and age of the pet, the source of and the age at acquisition, the length of the ownership, whether the respondent was the primary caregiver, presence of other household pets, and the current status of ownership (at follow-up surveys).

#### Owner-pet relationship

We used the Inclusion of Other in the Self (IOS) Scale [40], the Dog Owner Relationship Scale (DORS) [41], and the Cat-Owner Relationship Scale (CORS) [41] to quantify the perceived closeness between the pet and its owner.

The IOS scale consists of seven paired Venn diagrams, depicting different degrees of closeness that owners feel toward their pets. This resulted in a single score (1 to 7) with higher scores indicating closer owner-pet relationships.

The DORS and CORS, both are modified from the Monash Dog Owner Relationship Scale [42], consisting of a total of 28 and 26 (1: Disagree strongly to 5: Agree strongly) Likert-scale items, respectively. Both instruments measure three independent subscales: 1) perceived emotional closeness (10 and 11 items, respectively), 2) perceived costs (9 items each), and 3) owner-pet interaction (9 and 6 items, respectively). For each subscale, a mean score was computed, with higher scores indicating closer owner-pet relationships. Example items include statements such as “My dog (cat) helps me get through tough times” (for perceived emotional closeness) and “My dog (cat) costs too much money” (for perceived costs), and questions such as “How often do you kiss your dog (or cat)?” (for owner-pet interaction).

#### Perceived stress

We used the 10-item Perceived Stress Scale (PSS) [43] to quantify levels of stress. The PSS asks respondents to rate from “0” (never) to “4” (very often), how often they had certain feelings and thoughts (10 items) during the last month. Example questions include “How often have you been upset because of something that happened unexpectedly?” and “How often have you felt that you were unable to control the important things in your life?”. A total score (ranging from 0 to 40) was derived by summing the scores for all items, with higher scores indicating higher levels of stress.

#### Perceived loneliness

To quantify levels of loneliness, we used the 15-item Social and Emotional Loneliness Scale for Adults Short Version (SELSA-S) [44]. The SELSA-S is a modified version of the original SELSA consisting of 37 items and has been validated to measure comparable psychometric properties of bidimensional loneliness: 1) social loneliness and 2) emotional loneliness, as proposed by Weis in 1973 [45, 46]. Social loneliness results from a perceived lack of friendships or workplace relationships, whereas emotional loneliness is caused by deficiencies in intimate or family relationships. The SELSA-S consists of 15 Likert-scale items (each ranging from 1 to 7), measuring three subscales (5 items each): 1) social loneliness, 2) emotional loneliness—family, and 3) emotional loneliness—romantic. For each subscale, a mean score was computed, ranging from 1 to 7, with higher scores indicating higher levels of loneliness. Example items include “I don’t have any friends who share my views, but I wish I did” (for social loneliness), “I feel alone when I am with my family” (for emotional loneliness—family), and “I wish I had a more satisfying romantic relationship” (for emotional loneliness—romantic).

We administered the IOS scale, the DORS, the CORS, the PSS, the SELSA-S, and the questions measuring time-varying covariates (e.g., pet ownership status, other household pets, presence of other household members, housing, and COVID precautions and restrictions) at each data collection time point during the study period.

### Statistical analysis

Stata (*Release 17*. College Station, TX: StataCorp LLC) was used for all data analyses.

#### Temporal patterns of owner-pet relationship, stress, and loneliness

We used mixed linear regression models with random intercepts, to assess the changes in the owner-pet relationship, stress levels, and loneliness levels across the four phases of the COVID-19 pandemic within each of the three study groups. Each of the aforementioned measures (i.e., the IOS scale, the three subscales of the DORS or CORS, the PSS, and the three subscales of the SELSA-S) was separately modeled as the response variable. In each model, phase, pet ownership group, and the interaction between them were included as independent variables. The interaction was examined to assess whether the temporal patterns differed among the groups.

#### Effect of pet ownership on stress and loneliness

**Fig 1:**
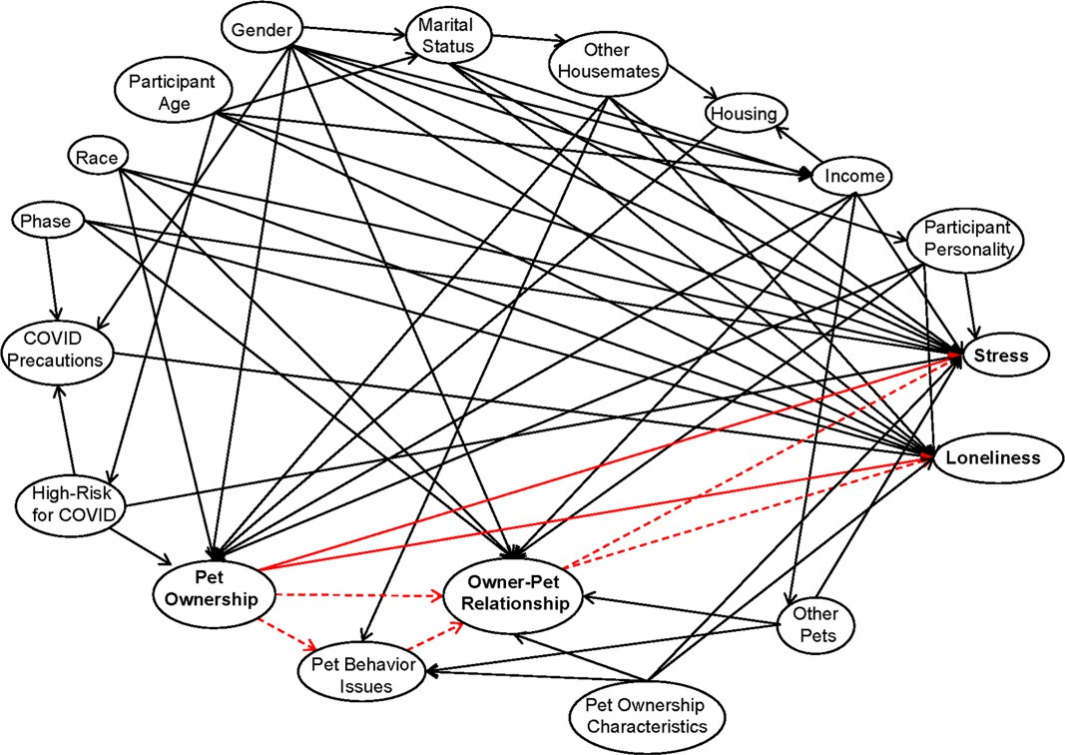
The directed acyclic graph depicts the study’s causal assumptions in the assessment of the effects of pet ownership on stress and loneliness. Red solid lines represent the direct effects and red dashed lines represent the indirect effects of Pet Ownership on Stress and Loneliness through Owner-Pet Relationship.

To assess the effect of pet ownership on stress and loneliness, we constructed a DAG to explicitly present our causal assumptions about pet ownership, owner-pet relationship, other covariates, and their causal relationships with each other and with stress and loneliness (Fig 1). An arrowed line connecting between two variables implied a direct causal relationship between them, with the direction of an arrowhead indicating which variable was a cause of the other. For example, according to the DAG, we assumed that Pet Ownership Characteristics was a common (direct) cause of Pet Behavior Issues, Owner-Pet Relationship, Stress, and Loneliness. We also assumed that Pet Ownership was associated with Stress and Loneliness through direct causal pathways and two different indirect causal pathways: one via Owner-Pet relationship and the other via Pet Behavior Issues and Owner-Pet Relationship.

We used the DAGitty program to build and analyze the DAG [47]. DAGitty implements the g-separation algorithm, which was used to identify minimally sufficient sets of confounders for adjustment. Based on our causal assumptions, the sufficient set of confounders was the same for both outcomes (stress and loneliness) and included Gender, High-Risk for COVID, Income, Other Housemates, Participant Personality, and Race.

The PSS score and the three subscale scores of the SELSA-S were modeled as the response variable in separate mixed linear models. In each model, pet ownership group and identified confounders were included as the independent variables. All identified confounders were included in the mixed linear models for adjustment without further covariate selection. In addition, the interaction between pet ownership group and phase was investigated in the initial model. If it was significant (i.e., p < 0.05), it was kept in the model and phase-specific adjusted effects were reported. Otherwise, the interaction term was dropped and pooled adjusted effects were reported. The interaction was examined to assess whether the effect of pet ownership, as quantified by the difference in the mean scores, varied across the phases of the pandemic.

#### Mediation effect of owner-pet relationship among pet owners

To assess whether the differences in the effect on stress and loneliness between dog and cat ownerships were mediated by owner-pet relationship, we partitioned the total effect of pet ownership into direct and indirect effects. As shown in the DAG (Fig 1), the total effects of pet ownership on stress and loneliness consisted of the direct effects (red solid lines) and the indirect (i.e., mediation) effects through owner-pet relationship (red dashed lines). Thus, we estimated the mediation effect of owner-pet relationship by subtracting the direct effect from the total effect.

The total and direct effects were quantified using the estimated regression coefficients of pet ownership (the cat group as reference) derived from the mixed linear regression models. The estimated regression coefficients represented the differences in the mean of the response variable (i.e., the PSS scores and the three subscale scores of the SELSA-S) comparing the dog to cat group. These coefficients were adjusted for the sufficient sets of confounders for estimating the total and direct effects identified using the g-separation algorithm based on our causal assumptions. The sufficient set of confounders used for estimating the total effects on stress and loneliness among pet owners was the same as the previously used set for estimating the effects of pet ownership, among all three groups, on these outcomes. Additional confounders for adjustment in the estimation of the direct effect on stress included Other Pets, Pet Ownership Characteristics, Owner-Pet Relationship, and Phase, while additional confounders for estimating the direct effect on loneliness were Pet Ownership Characteristics, Owner-Pet Relationship, and Phase. All owner-pet relationship measures (i.e., IOS scale, owner-pet interaction subscale, emotional closeness subscale, and perceived costs subscale) were included as confounders in the direct effect models.

We further quantified the magnitude of the mediation effect of owner-pet relationship using the percent change in the estimated regression coefficients between the total and direct effect models (Eq 1).


$$\left[\left(Total\;effect-Direct\;effect\right)/Total\;effect\right]\times 100\%$$ (1)

Whenever the missing values of any confounder in a minimally sufficient set chosen for control caused a reduction in the analytical sample size by 20% or more, we applied sequential imputation with the assumption of monotone-missing pattern in Stata [48]. Thirty sets of multiple imputations were generated and used to derive summary estimates and their corresponding 95% confidence intervals.

## Results

A total of 4,237 participants (657 non-pet owners, 1,761 dog owners, and 1,819 cat owners) completed between one and six surveys during the study period. Among pet owners, 1,186 had dogs only, 1,128 had cats only, and 1,266 had both dogs and cats. Table 1 summarizes the information about the study cohorts recruited at different survey time points and the overall retention rates at the end of the study period.

**Table 1:** Study cohorts, times of recruitment, and overall retention rates.

Cohort	Time of recruitment (Month, Year)	Number of participants	Retention rate
1	June, 2020	1,573	27%
2	January, 2021	1,269	37%
3	April, 2021	235	37%
4	August, 2021	631	58%
5	December, 2021	529	100%
**Total**		4,237	44%

Overall retention rates are computed at the last survey in December 2021

The demographic information of the participants and the characteristics of the pets are summarized in Table 2.

**Table 2:** Descriptive statistics of the study participants by their pet ownership status (No pet, Dog, and Cat).

	No pet	Dog	Cat
**Demographics of participants**			
Age at the initial survey	37 (19–71)	37 (18–79)	36 (18–72)
Gender			
Female	269	850	907
Male	381	888	845
Non-binary	<5	12	18
Race			
Asian	49	43	34
Black or African American	52	41	43
White	213	738	756
Other	21	44	43
**Characteristics of pets**			
Age at the initial survey			
<6 months		6	12
6 months–2 years		186	212
3–10 years		476	617
11–15 years		404	169
16 years and older		20	67
Sex			
Female, intact		424	248
Female, spayed		506	598
Male, intact		286	316
Male, neutered		539	611
Source of acquisition			
Birth of a family pet		307	352
Breeder		573	244
Shelter/Rescue		634	775
Other		167	340
Age of acquisition			
<2 months		383	476
2–5 months		609	576
6 months–1 year		345	331
2–7 years		377	280
8 years and older		41	77
Primary caregiver			
Yes		1,630	1,648
No		121	123

Only variables that do not vary over time are reported.

Age of participant is reported as median (range); all other variables are reported as frequency counts

The total number for each variable may vary due to missing values or respondents declining to answer

### Temporal patterns of owner-pet relationship

**Fig 2:**
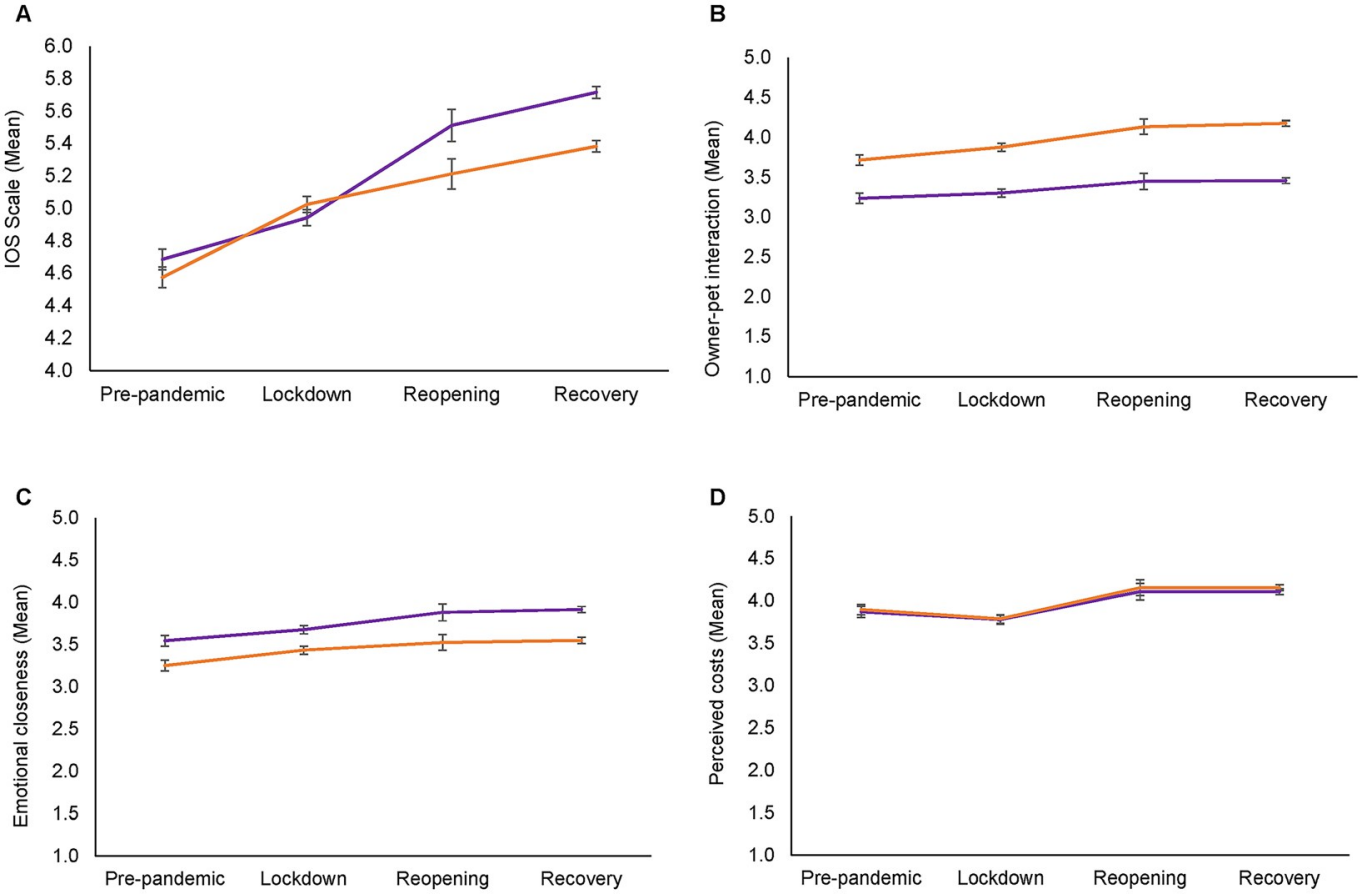
Temporal patterns of owner-pet relationship across the four phases of the COVID-19 pandemic. Owner-pet relationship is measured using A) the Inclusion of Other in the Self (IOS) scale, B) the owner-pet interaction, C) emotional closeness, and D) perceived costs subscales. Higher scores indicate closer attachment to the pet. The mean scores and standard errors (error bars) are derived from mixed linear models (N = 3,579). Purple line: dog ownership; Orange line: cat ownership.

Although the temporal changes from the pre-pandemic to recovery phases were all small, an overall positive trend was observed in all owner-pet relationship measures in both dog and cat groups (Fig 2). The mean IOS score increased from 4.7 (95% CI 4.6, 4.8) in the pre-pandemic phase to 5.7 (95% CI 5.6, 5.8) in the recovery phase for the dog group, and from 4.6 (95% CI 4.4, 4.7) in the pre-pandemic to 5.4 (95% CI 5.3, 5.5) in the recovery phase for the cat group (Fig 2A). The examination of the interaction between group and phase showed significant differences in the temporal patterns between the dog and cat groups (p<0.001). The largest difference in the temporal patterns of the IOS scale between the two groups was observed during the change from the lockdown to reopening, during which the dog group showed a larger increase in the mean IOS score (0.57, 95% CI 0.33, 0.81) than the cat group (0.19, 95% CI -0.02, 0.40). From the pre-pandemic to recovery phases, only subtle changes were observed in the mean scores of the owner-pet interaction and the perceived emotional closeness subscales, respectively, for the dog (increased by 0.23 and 0.37) and cat (increased by 0.46 and 0.30) groups (Fig 2B and 2C). The mean perceived costs scores were lowest in the lockdown phase in both pet groups (Dog: 3.8 and Cat: 3.8), indicating the least attachment to the pet due to the concerns related to the cost of having a pet, and were highest in the reopening (Dog: 4.1 and Cat: 4.2) and recovery (Dog: 4.1, Cat: 4.2) phases (Fig 2C).

### Temporal patterns of stress

**Fig 3:**
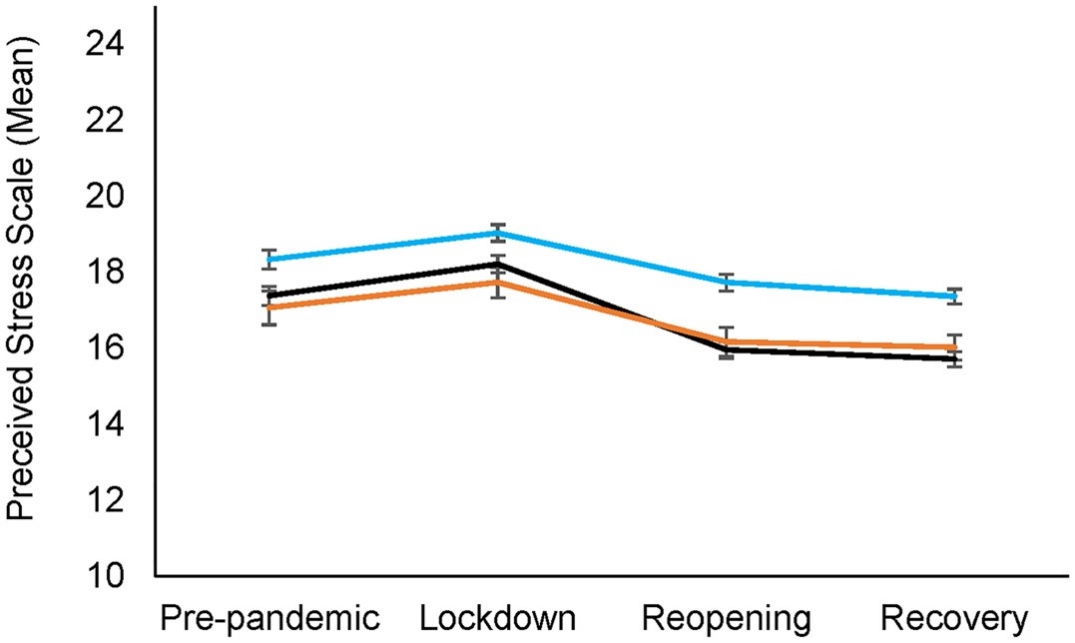
Temporal patterns of stress level by pet ownership groups across the four phases of the COVID-19 pandemic. Stress level is measured using the Perceived Stress Scale (PSS), with high scores indicating higher degrees of stress. The mean PSS scores and standard errors (error bars) are derived from a mixed linear model (N = 4,235). Black line: dog group; Blue line: cat group; Orange line: no-pet group.

The temporal changes in the mean PSS score were small and similar across all three groups with the peak levels of stress during the lockdown phase and lowest levels during the recovery phase (Fig 3). The dog group had the largest reduction in the mean PSS score from the lockdown phase to the recovery phase (-2.5; 95% CI -3.1, -1.9), whereas the no-pet group (-1.7, 95% CI -2.8, -0.61) and the cat group (-1.7, 95% CI -2.2, -1.1) were comparable. Cat owners consistently reported the highest stress levels in all phases.

### Temporal patterns of loneliness

**Fig 4:**
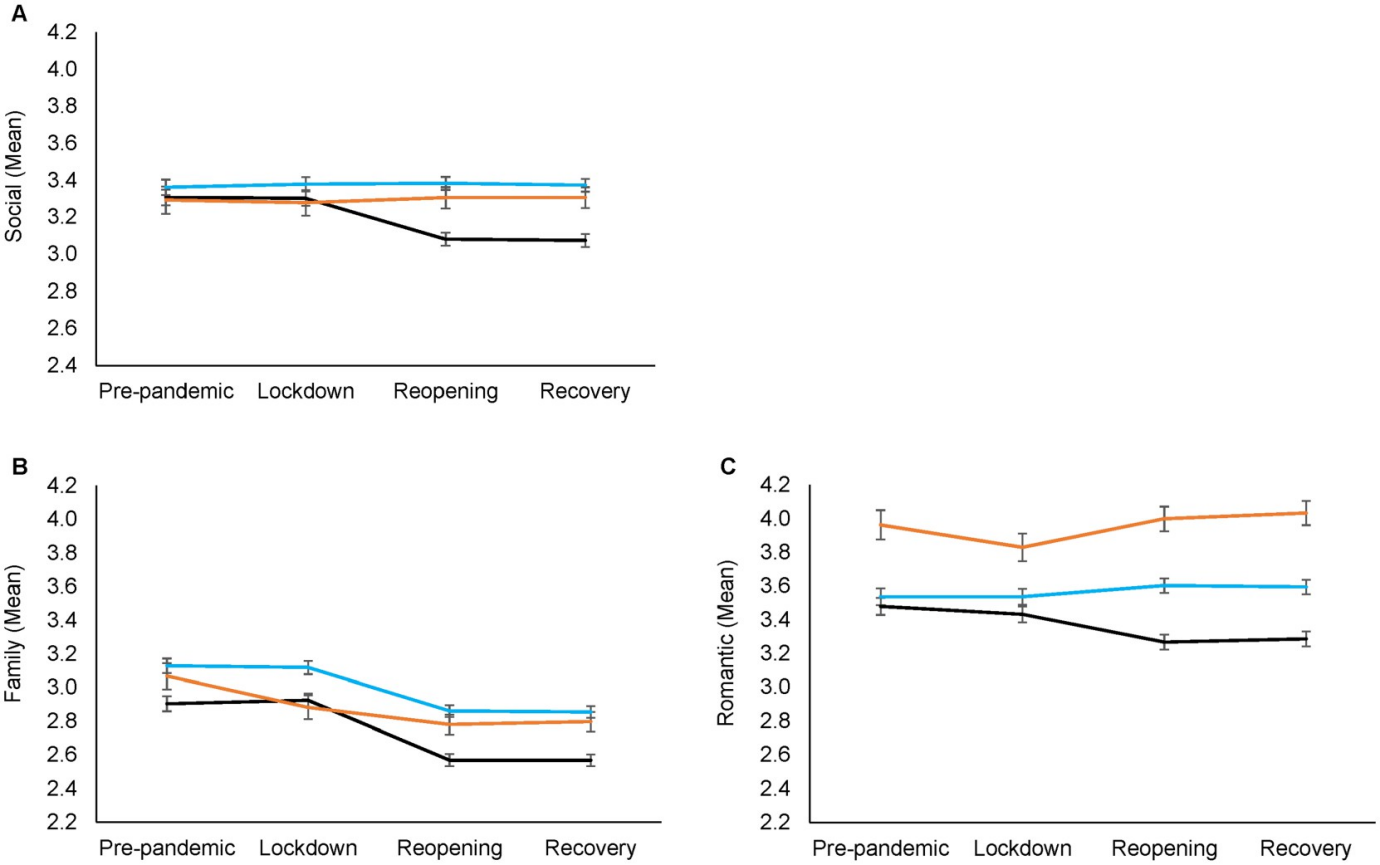
Temporal patterns of loneliness levels by pet ownership groups across the four phases of the COVID-19 pandemic. The levels of loneliness are measured using the Social and Emotional Loneliness Scale for Adults Short Version (SELSA-S) with three subscales: A) social loneliness (Social), B) family emotional loneliness (Family), and C) romantic emotional loneliness (Romantic). Higher scores indicate higher degrees of loneliness. The mean scores and standard errors (error bars) are derived from mixed linear models (N = 4,235). Black line: dog group; Blue line: cat group; Orange line: no-pet group.

Temporal patterns in the level of loneliness varied across the study groups, showing that the dog group consistently showed the largest reduction in all three loneliness subscales between lockdown and recovery phases, while the cat group and no-pet group were similar (Fig 4). The difference between the dog group and the other two groups was most profound in the social loneliness subscale, which showed a decline from the lockdown phase to the reopening phase with a change in the mean score of -0.22 (95% CI -0.31, -1.3), whereas there was almost no change in the cat and no-pet groups (Fig 4A). Both dog and cat groups had similar temporal patterns for the family emotional subscale, which showed a decrease in the mean score from the pre-pandemic phase to the reopening phase (Dog: -0.34 and Cat: -0.27). The mean family emotional loneliness score for the no-pet group decreased from the pre-pandemic to reopening phases but leveled off from the reopening phase to the recovery phase. Temporal changes in the mean romantic emotional loneliness score were very small in all groups. Both cat and no-pet groups showed slight increases in the mean romantic emotional loneliness score, whereas the dog group showed a decrease in the mean score of -0.21 (95% CI -0.31, -0.11) from the pre-pandemic phase to the reopening phase.

### Effect of pet ownership on stress and loneliness

After controlling for the confounders, the interaction between group and phase was not significant in the model assessing the effect of pet ownership on stress, thus, the pooled effects were reported. The adjusted results showed that the cat group had a higher level of stress compared to the dog group (difference in the mean PSS score of 0.99 (95% CI 0.54, 1.4)) and the no-pet group (difference in the mean PSS score of 1.3 (95% CI 0.66, 1.9)).

Because the interactions between group and phase were significant in all three perceived loneliness subscales after adjusting for the confounders, comparisons between groups were done in each of the phases. Levels of social loneliness were similar among the three groups in the pre-pandemic and lockdown phases but, the cat group had higher levels of social loneliness during the reopening and recovery phases compared to the other two groups (Table 3). For the family emotional loneliness subscale, the dog group had the lowest levels across all the phases, whereas the cat group had highest levels from the lockdown phase onwards. In contrast, the no-pet group had the highest levels of romantic emotional loneliness in all four phases of the pandemic. The dog group, again, had the lowest levels among the three groups.

**Table 3:** Differences in the mean (95% confidence interval) loneliness scores comparing dog, cat, and no-pet groups in each of the four phases of the COVID-19 pandemic. Social loneliness (Social) and emotional loneliness due to the deficits in family relationships (Family) and intimate relationships (Romantic) are quantified using the Social and Emotional Loneliness Scale for Adults Short Version, each range from 1 to 7 with higher scores indicating higher degrees of loneliness. N = 4,166.

	Pandemic phases			
	Pre-pandemic	Lockdown	Reopening	Recovery
Social				
Dog vs. No-pet	-0.09 (-0.27, 0.08)	-0.09 (-0.26, 0.07)	-0.05 (-0.21, 0.11)	-0.09 (-0.22, 0.04)
Cat vs. No-pet	-0.04 (-0.21, 0.14)	-0.01 (-0.18, 0.15)	0.23 (0.07, 0.38)	0.14 (0.003, 0.27)
Dog vs. Cat	-0.06 (-0.18, 0.06)	-0.08 (-0.19, 0.03)	-0.28 (-0.38, -0.17)	-0.22 (-0.32, -0.13)
Family				
Dog vs. No-pet	-0.26 (-0.43, -0.08)	-0.05 (-0.22, 0.12)	-0.05 (-0.20, 0.11)	-0.11 (-0.24, 0.03)
Cat vs. No-pet	-0.03 (-0.21, 0.15)	0.14 (-0.02, 0.31)	0.20 (0.04,0.35)	0.12 (-0.01, 0.25)
Dog vs. Cat	-0.23 (-0.34, -0.11)	-0.19 (-0.30, -0.08)	-0.25 (-0.36, -0.14)	-0.23 (-0.32, -0.13)
Romantic				
Dog vs. No-pet	-0.49 (-0.70, -0.27)	-0.41 (-0.61, -0.21)	-0.61 (-0.80, -0.42)	-0.70 (-0.87, -0.53)
Cat vs. No-pet	-0.40 (-0.62, -0.19)	-0.27 (-0.47, -0.07)	-0.35 (-0.54, -0.16)	-0.46 (-0.63, -0.30)
Dog vs. Cat	-0.08 (-0.22, 0.06)	-0.14 (-0.27, -0.002)	-0.26 (-0.39, -0.13)	-0.23 (-0.35, -0.11)

### Mediation effect of owner-pet relationship on stress and loneliness

The results showed weak to moderate mediation effects of owner-pet relationship on both stress and loneliness that consistently reduced the observed differences between the dog and cat groups (Table 4).

**Table 4:** Comparison between the total and direct effect models of pet ownership type (dog vs. cat ownership) on stress, social loneliness (Loneliness—Social), and emotional loneliness due to the deficits in family relationships (Loneliness—Family) and intimate relationships (Loneliness—Romantic).

	Total effect (95% CI)	Direct effect (95% CI)	Difference (% change)
Stress (N = 3,508)	-0.92 (-1.4, -0.47)	-1.1 (-1.5, -0.60)	0.18 (-20%)
Loneliness—Social (N = 3,503)	-0.17 (-0.26, -0.08)	-0.19 (-0.27, -0.10)	0.02 (-12%)
Loneliness—Family (N = 3,502)	-0.21 (-0.30, -0.12)	-0.27 (-0.36, -0.18)	0.06 (-29%)
Loneliness—Romantic (N = 3,501)	-0.19 (-0.30, -0.08)	-0.21 (-0.32, -0.09)	0.02 (-11%)

Regression coefficients (cat ownership as the reference) and 95% confidence intervals (CI) derived from mixed linear models are reported.

The confounders included in the total effect models for both stress and loneliness are Gender, High-Risk for COVID, Income, Other Housemates, Participant Personality, and Race.

The direct effect models contain all the confounders in the total effect models plus Other Pets, Pet Ownership Characteristics, Owner-Pet Relationship, and Phase for stress, and Pet Ownership Characteristics, Owner-Pet Relationship, and Phase for loneliness.

Difference = Total effect − Direct effect

% change = (Total effect − Direct effect) / Total effect × 100%

## Discussion

In this study, we investigated the temporal changes in the owner-pet relationship as well as levels of stress and loneliness from February 2020 through 2021. To our best knowledge, this is the first longitudinal study to examine the temporal patterns of these variables during the COVID-19 pandemic. The results showed that the owners reported a closer relationship with their pet dogs and cats as the pandemic progressed, with the exception of perceived closeness due to cost-related factors during the lockdown phase of the pandemic. In this instance, there was a slight decrease in the mean score indicating a slight distancing. We also observed changes in self-reported stress and loneliness levels during the pandemic as well as the differences among dog, cat, and non-pet owners. The levels of stress were the highest during the lockdown phase in all three study groups but quickly reduced thereafter. The dog group consistently experienced the greatest decreases in social and emotional loneliness in the reopening and recovery phases, whereas only subtle temporal changes were observed in the cat and no-pet groups. After adjusting for confounders, our results did not support the hypothesis that pet ownership buffered stress and loneliness during the pandemic. On the contrary, cat owners reported higher degrees of stress, social loneliness, and family emotional loneliness when compared to dog and non-pet owners. Further investigation revealed that the differences between dog and cat ownerships might be partially explained by the owner-pet relationship.

Contrary to our hypothesis that the sudden disruption and changes in routines at the beginning of the COVID-19 pandemic would negatively affect the owner-pet relationship, our results showed an overall positive, though subtle, trend in all owner-pet relationship measurements in both dog and cat owners since the beginning of the pandemic. These findings may be explained by owners spending more time at home and being more socially isolated from non-family members during the pandemic, resulting in a feeling of stronger attachment to their pets. Support for these results is evident in the increased demands for pet dogs and cats during the early pandemic period [8, 49–52]. It is important to note that the increasing trend in the closeness of the owner-pet relationship did not stop even after two years subsequent to the start of the pandemic except for in the case of the perceived costs subscale, indicating the resilience of the bonds between the owners and their pets against adversity once established [24, 30, 32, 53]. The majority (91%) of the participants in this study had the pets for one year or longer, which further supports this premise.

The initial decrease in the perceived costs subscale for the owner-pet relationship during the lockdown phase aligns with our hypothesis regarding the adverse impact of the pandemic on the financial burden of having a pet. However, the decline was reversed quickly and returned to levels comparable to those of the pre-pandemic phase after the lockdown phase. These findings are similar to the findings in a previous study of the impacts of the 2008–9 economic recession on the owner-pet relationship [32]. In that study, the authors did not observe an increase in the relinquishment of pet dogs and cats in a Chicago animal shelter, although a decrease in dog adoptions was observed during that period. A survey of U.S. residents in May 2021 also did not find a substantial increase in pet rehoming during the pandemic [24]. Some risk factors for pet rehoming identified in that study, however, did suggest there were concerns related to the cost of having a pet, such as access to affordable housing, pet supplies, and veterinary care. Although these risk factors may not be unique to the pandemic, the findings provided evidence-based guidance to relevant agencies in providing pet owners support during the pandemic.

We observed similar temporal patterns in all owner-pet relationship measures between the dog and cat groups. Overall, the dog group had a slightly larger increase in the mean scores of the IOS scale and the perceived emotional closeness subscale from the pre-pandemic phase to the recovery phase. The only exception was the owner-pet interaction subscale, for which the cat group showed a slightly greater increase than the dog group. The owner-pet interaction subscale of the DORS and CORS contained different items to better reflect the differences in the common activities the owners would have with their pet dogs and cats, respectively. The discrepancy in this subscale may be due to the differences in the impact of lockdown on the owner-dog and owner-cat interactions. For example, certain types of owner-dog interactions, such as taking the dog in the car and taking the dog to visit others, might have been reduced more during the lockdown phase than some types of owner-cat interaction. We also observed the difference in the changes in the IOS scale from the lockdown phase to the reopening phase, during which the dog group showed a larger increase in the closeness of owner-pet relationship than the cat group. The IOS scale contains a series of paired Venn diagrams, portraying different degrees of the closeness the owners feel toward the pets and results in a single score. Thus, it may not capture the complex and multifaceted properties of the owner-pet relationship as measured in the three subscales of the DORS and CORS. A larger increase in the mean IOS score in the dog group than in the cat group may reflect the difference in the perceptions of the graphical presentation of closeness between dog and cat owners. We noted that, however, all the observed differences in temporal patterns between the dog and cat groups were small.

The dog group consistently showed the largest decreases in levels of stress and loneliness during the reopening and recovery phases, whereas the cat group had similar temporal patterns as those of the no-pet group. The most profound difference was in social loneliness, in which the dog group showed a decrease from the lockdown phase to the reopening phase, whereas both the cat and no-pet groups did not show any temporal changes. However, after controlling for confounders, the results did not support a mitigating effect of pet ownership on stress and loneliness. The no-pet group, on average, showed the lowest levels of stress and the cat group the highest in all four phases of the pandemic. The cat group also showed higher levels of social and family emotional loneliness than the other two groups during the reopening and recovery phases of the pandemic. These findings are consistent with the results of some previous studies [12, 54–56] but not with others [14, 57, 58]. The conflicting findings across studies may be due to differences in study methodology (e.g., quantitative vs. qualitative and/or approaches to confounder adjustment between quantitative studies) and the differences in the scales used to quantify mental health and well-being [59]. A systematic review of the effect of pet ownership on loneliness and social isolation noted that a majority of the studies that suggested a mitigation effect of pet ownership on mental health during the COVID-19 pandemic were qualitative studies, whereas the quantitative evidence was much less consistent [19]. A similar disparity between quantitative and qualitative evidence was also observed in a recently published mixed-methods study [15]. In this study, the pet owners reported positive influences of their pets during the pandemic but the quantitative data provided no evidence of a link between pet ownership and loneliness. Our finding that pet owners had a higher degree of stress than non-pet owners may be explained by additional challenges and uncertainties, such as financial burden, veterinary care access, and disease transmission, which pet owners face during a crisis [20, 25]. We note that the non-pet group consistently had the lowest degree of stress even before the pandemic, and therefore cannot rule out the fact that in this cohort of individuals non-pet owners might be systematically less stressed than pet owners. Nonetheless, adjusted differences in the mean PSS score between groups were all very small relatively to the range of the PSS (0 to 40).

Also contradicting our presumption that pet ownership would have a greater mitigating effect on social loneliness, the only construct in which pet ownership consistently showed a lower level of loneliness was the romantic emotional loneliness. One possible explanation for finding only subtle differences in the perceived social loneliness between the pet and no-pet groups was that because social interactions were greatly reduced during the pandemic, the benefits of pets facilitating owners’ social connections were limited. On the other hand, both family and romantic emotional loneliness subscales of the SELSA-S measure loneliness resulting from conflicts between intimate partners and family members, respectively [44]. Because people spent more time at home during the pandemic, conflicts between intimate partners or family members living in the same household were more likely to occur. Pets might have served as a buffer in those conflicts to prevent the elevation of emotional loneliness. However, pet ownership only showed a mitigating effect on the romantic emotional loneliness but not on the family emotional loneliness. This finding might reflect the differences between these two types of relationships, and that pet ownership had a stronger effect on mitigating the deficits of intimate relationships than family relationships. However, we did not measure participants’ intimate relationships or family structures to directly examine these notions. In addition, the observed differences between the study groups might be due to the differences in the respondents’ interpretations of the items of the SELSA-S. Nevertheless, our use of the SELSA-S revealed how pet ownership affected the three loneliness constructs differently.

Overall, the cat group in this study tended to have the highest levels of perceived stress and loneliness. A similar finding was also reported in a study of an Australian population who lived alone [56]. In that study, the authors found that cat owners were less mindful (defined as able to attend to the present) than non-pet owners, and pet interactions did not account for levels of loneliness. We also found only weak to moderate mediation effects of owner-pet relationship, despite using different measures to quantify owner-relationship and different approaches to estimate its effects. Interestingly, our results showed that the direction of the mediation effects consistently suggested a reduction in the differences between dog and cat ownerships after considering owner-pet relationship.

Clements et al. investigated the links between companion animal type and level of owner-pet interactions and loneliness during the pandemic using both quantitative and qualitative methods [15]. They found that a greater level of dog-owner interaction was associated with a higher level of loneliness. They argued that a higher level of loneliness might result in a greater level of dog-owner interaction. This is opposite to our assumption that pet ownership precedes perceived stress and loneliness (Fig 1). These opposing viewpoints highlight the importance of causal assumptions in making inferences from observational studies [26]. Our use of DAGs to explicitly communicate our underlying causal assumptions not only explains our selection of confounders for adjustment but also opens the opportunity for further discussion. Although the majority of the pet owners in this study owned their pets for more than one year, the association between the owner-pet relationship and perceived stress and loneliness may well be bi-directional. Despite the longitudinal nature of the study data, the data on owner-pet relationships and perceived stress and loneliness were collected at the same time in each survey. Thus, the temporal relationship between the two may not be distinguishable in the data collected.

This study had several additional limitations that may affect the validity of the findings. First, the recruitment of our first cohort started in June 2020. Thus, the data on the pre-pandemic phase (February 2020) and part of the lockdown (April 2020) phase were collected retrospectively. This was necessary as the study could commence only after the official declaration of the pandemic (i.e., in March 2020). Participants’ recall of earlier perceptions in February and April might have been affected by their current perceptions while completing the survey in June 2020. Bearing this limitation in mind, we emphasize the overall temporal patterns rather than direct comparisons of other phases to the pre-pandemic phase. Second, most of the study participants only completed three or fewer surveys (maximum of six surveys) during the study period. Although the overall retention rate of this study was similar to previous investigations [60, 61], the number of the initial cohort members (recruited in June 2020) who did not participate in the second survey in September 2020 was greater than expected. Because the information on several identified confounders, including Participant Personality, Race, and Income, was not collected in the first survey, we implemented imputation to replace the missing values. In order to get reliable estimates, we generated 30 random imputed datasets and analyzed them to derive summary estimates. Nonetheless, we did not quantify the potential impact of the imputations. Third, once a study participant reported a change in the pet-ownership status, the participant was censored and no further follow-up data was collected. However, the data collected in their last completed survey were included in the analysis. We specified in the questions asking them to report the data reflecting the period before the change in the pet-ownership status. Fourth, the study data were collected through surveys and only measured the subjective perceptions of the participants on the closeness of the pet-ownership, and the levels of stress and loneliness. Previous studies have shown discrepancies between subjective and physiological measures of these parameters [62, 63]. Our findings also suggested the differences in the interpretations of the scale items among dog owners, cat owners, and non-pet owners. Finally, we only presented and examined one set of causal assumptions. Different causal assumptions may lead to different sets of confounders being identified for adjustment. The purpose of explicitly presenting our causal assumptions is to provide sufficient information to readers to allow for transparent discussion, if desired.

Notwithstanding limitations, this study highlights the dynamic effects of the COVID-19 pandemic on owner-pet relationships and perceived stress and loneliness. It also shows the complexity of the association between pet ownership and mental health, partially mediated through owner-pet relationship. Despite the additional challenges the pet owners may face during the pandemic, they felt closer to their pets throughout the study period. The dog group consistently showed the largest decreases in the levels of stress and loneliness during the reopening and recovery phases. However, after adjusting for the identified confounders based on our causal assumptions, the results did not support the hypothesis that pet ownership mitigated stress and loneliness during the pandemic. On the contrary, the cat group tended to show higher levels of stress and loneliness. Our further assessment revealed that the observed differences between dog and cat ownerships might be partially explained by the mediation effect of owner-pet relationship. The differences between them were reduced after considering owner-pet relationship. We will continue collecting follow-up data through 2023, and expect to see further changes in owner-pet relationships and stress and loneliness after the period presented.
